# Response to letter titled ‘Reduction of HbA1c in patients with type 2 diabetes following duodenal mucosal resurfacing: could other factors be at play?’

**DOI:** 10.1136/gutjnl-2020-321170

**Published:** 2020-03-30

**Authors:** JJGHM Bergman

**Affiliations:** Department of Gastroenterology, Academic Medical Center, Amsterdam 1105 AZ, The Netherlands

**Keywords:** diabetes mellitus, endoscopic procedures, duodenal mucosa

We agree with Johnston and colleagues^1^that it is important to consider potential confounding factors, such as diet and exercise, when interpreting the results of our study.^2^

Our study did not include specific dietary counselling of patients, and there was no concerted effort to have patients adhere to a specific hypocaloric regimen. During follow-up visits, patients underwent per protocol (PP) dietary counselling based on standard clinical practice guidelines. These guidelines emphasise the relation of carbohydrate intake, glycaemic index and blood glucose control. Taking into account that patients willing to participate in a study are generally more motivated to make behavioural changes, it is possible that the subjects in this study reduced their total carbohydrate intake during this study. However, since we did not record specific data on dietary intake, we cannot address this issue.

Regarding the food preference after bariatric procedures: indeed there is some evidence of food preference changes in patients who have undergone gastric bypass surgery. However, this evidence is large based on indirect measurements, mostly patients’ self-reporting.^3^ More recent studies, in which changes in food preferences were directly assessed, contradict previous findings and/or warn for over interpreting self-reported changes in food preference.^4^

The weight loss observed in this study might also be a confounding factor. However, the total weight loss of ~2.3 kg observed occurred in the first 4 weeks of the study when patients were on a liquid diet transitioning into solid foods over 2 weeks postprocedure, after which weight stabilised. In our opinion, the limited weight loss and its course over time do not suggest that this is a major confounding factor in our study.

Johnston and colleagues inquire if the baseline characteristics of the PP population are comparable to those of the intention-to-treat (ITT) population. The online supplementary table indicates that the baseline characteristics were comparable.

10.1136/gutjnl-2020-321170.supp1 Supplementary data

In our opinion, it is not accurate to use the nine excluded patients as control group to compare efficacy to the ITT population: all nine excluded patients underwent at least a partial duodenal ablation (two patients underwent two ablations; one patient had three ablations; and six patients had four ablations) and therefore will have had some therapeutic effect. Fortunately, data of a sham-controlled randomised study will come available shortly.

Our team is eager to elucidate the underlying mechanism how duodenal ablation improves glycaemic and metabolic health in patients with type 2 diabetes. The considerations raised by Johnston and colleagues will be taken into account when designing new studies of duodenal ablation in patients with type 2 diabetes.
