# Supplementary material for: Response to letter titled ‘Reduction of HbA1c in patients with type 2 diabetes following duodenal mucosal resurfacing: could other factors be at play?’
Source: Gut. 2020 Mar 30;70(1):218. doi: 10.1136/gutjnl-2020-321170 (PMC7788196; doi:10.1136/gutjnl-2020-321170)
Supplement: Supplementary data [file gutjnl-2020-321170supp001.pdf]

Supplementary table

Baseline Characteristics			
**Characteristics**	**ITT N=46**	**PP N=36**	**Excluded from PP N=10**
Age (years)			
Mean ± SD (N)	55.1 ± 8.4 (46)	55.7 ± 8.0 (36)	53.0 ± 9.9 (10)
Median (Min, Max)	55.0 (31.0, 69.0)	56.5 (31.0, 69.0)	54.0 (32.0, 67.0)
Sex [% (n/N)]			
Male	63.0% (29/46)	63.9% (23/36)	60.0% (6/10)
Female	37.0% (17/46)	36.1% (13/36)	40.0% (4/10)
Height (cm)[1]			
Mean ± SD (N)	170.8 ± 7.2 (46)	170.5 ± 7.3 (36)	171.9 ± 7.1 (10)
Median (Min, Max)	172.0 (156.0, 187.5)	172.0 (156.0, 187.5)	172.5 (160.0, 181.0)
Weight (kg)			
Mean ± SD (N)	90.3 ± 13.1 (42)	88.9 ± 11.3 (33)	95.2 ± 18.4 (9)
Median (Min, Max)	90.0 (59.0, 116.0)	87.0 (64.0, 115.0)	92.0 (59.0, 116.0)
BMI (kg/m^2)[1]			
Mean ± SD (N)	31.6 ± 4.4 (46)	31.3 ± 4.2 (36)	32.6 ± 5.2 (10)
Median (Min, Max)	30.8 (25.0, 39.6)	30.7 (25.0, 39.6)	32.6 (25.1, 39.6)
Systolic Blood Pressure (mmHg)[1]			
Mean ± SD (N)	137.4 ± 18.2 (46)	137.1 ± 17.2 (36)	138.6 ± 22.4 (10)
Median (Min, Max)	135.0 (110.0, 190.0)	135.0 (110.0, 179.0)	135.0 (110.0, 190.0)
Diastolic Blood Pressure (mmHg)[1]			
Mean ± SD (N)	85.2 ± 9.4 (46)	84.7 ± 9.4 (36)	86.9 ± 9.4 (10)
Median (Min, Max)	84.0 (60.0, 102.0)	82.5 (60.0, 102.0)	90.0 (70.0, 100.0)
Duration of T2D (years)			
Mean ± SD (N)	6.0 ± 2.9 (46)	6.1 ± 2.7 (36)	5.7 ± 3.6 (10)
Median (Min, Max)	6.4 (0.1, 11.5)	6.5 (0.1, 9.8)	6.0 (0.9, 11.5)
HbA1c (%)			
Mean ± SD (N)	8.6 ± 0.8 (46)	8.4 ± 0.7 (36)	9.3 ± 1.1 (10)
Median (Min, Max)	8.4 (7.5, 11.0)	8.3 (7.5, 9.8)	9.1 (7.5, 11.0)
Fasting Plasma Glucose (mg/dL)			
Mean ± SD (N)	195.6 ± 48.4 (44)	195.2 ± 42.1 (35)	197.0 ± 71.1 (9)

Baseline Characteristics			
**Characteristics**	**ITT N=46**	**PP N=36**	**Excluded from PP N=10**
Median (Min, Max)	195.5 (85.0, 320.7)	195.0 (114.0, 274.0)	200.0 (85.0, 320.7)
Total Cholesterol (mg/dL)			
Mean ± SD (N)	174.3 ± 38.7 (46)	173.1 ± 38.1 (36)	178.3 ± 42.7 (10)
Median (Min, Max)	169.5 (98.2, 280.0)	169.5 (98.2, 280.0)	170.1 (131.5, 267.0)
LDL-C (mg/dL)			
Mean ± SD (N)	99.0 ± 32.8 (46)	99.2 ± 30.0 (36)	98.3 ± 43.2 (10)
Median (Min, Max)	92.0 (23.2, 171.0)	92.0 (48.3, 171.0)	95.0 (23.2, 170.0)
HDL-C (mg/dL)			
Mean ± SD (N)	44.2 ± 9.9 (46)	43.8 ± 10.4 (36)	45.7 ± 8.1 (10)
Median (Min, Max)	44.5 (24.0, 67.0)	42.5 (24.0, 67.0)	45.5 (30.9, 58.0)
Triglycerides (mg/dL)			
Mean ± SD (N)	189.9 ± 113.6 (46)	187.2 ± 120.8 (36)	199.5 ± 87.5 (10)
Median (Min, Max)	160.2 (52.0, 670.0)	154.5 (52.0, 670.0)	192.0 (77.9, 398.6)
Oral Anti-Diabetic Medications at Screening [%(n/N)]			
Metformin	91.3% (42/46)	91.7% (33/36)	90.0% (9/10)
Sulfonylurea	30.4% (14/46)	25.0% (9/36)	50.0% (5/10)
Region			
Europe	82.6% (38/46)	86.1% (31/36)	70.0% (7/10)
South America	17.4% (8/46)	13.9% (5/36)	30.0% (3/10)

Note: If baseline value was not available, then used screening value.
